# Selections

**Published:** 1817-10

**Authors:** 


### PART III.

### SELECTIONS.

—— Factorum est copia nobis.

### FOREIGN WORKS.

### PATHOLOGY and PRACTICE.—*Rupture of the Diaphragm.*

This fatal accident is probably a more frequent effect of external violence, than, from the general silence of writers on pathology respecting it, we might be led to imagine. A very luminous and interesting account of it has recently been published, by the celebrated French surgeon Percy, in a work to which we shall have constant occasion to refer in tracing our monthly sketch of the medical literature and practice of the continent.* We proceed briefly to analyze it.

The diaphragm, like all the other organs, is subject to malformation, irregularity of situation, and every variety of morbid affection. It has been found by Morgagni and others at the superior part of the chest, in persons who have died of suffocation from this singular cause. Notwithstanding its importance, this muscle is sometimes wanting. Diemerbroeck found both it and the mediastinum deficient in a child of seven years, who exhibited no other symptom but a congenital cough. Inflammation, abscess, ulceration, gangrene, and tumours of the diaphragm have also been discovered in subjects supposed to have perished from very different diseases. Pus, formed in the liver, may make a way through it to the chest; or it may give passage to effused fluids from the latter cavity into the abdomen. When it has been penetrated by a sharp instrument or by ulceration, the floating viscera of the abdomen are almost always protruded into the thorax by the irresistible contraction of the abdominal muscles. Kirschbaum, in a work printed at Strasburgh in 1749, has collected nearly all the cases of this curious transmigration, known in his time. Since then, they have been too commonly observed to require particular citation. It has frequently happened, that, when the diaphragm has been pierced by a sharp sword or small musket-ball, a portion, or the whole, of the stomach, with more or less of intestine, has escaped through into the thorax. Ambrose Paré is one of the first writers by whom this occurrence has been recorded. The accident may

* *Dictionnaire des Sciences Médicales,* tom. ix. Article *Diaphragme.*

also take place by the œsophageal orifice of the diaphragm, when anywise it has become much enlarged. Schrober mentions the case of a man who was the subject of scirrhous liver, and died from gangrene, the consequence of frequent intoxication. On dissection, the stomach, duodenum, jejunum, and part of the ileum, were found confusedly situated in the thorax, into which they had passed through the dilated œsophageal opening of the diaphragm. By the same orifice, Fanton discovered the stomach and a portion of the omentum to have escaped in a young man, who, in consequence of a sword wound in the epigastrium, had been, for a year, subject to colics and vomiting, and had ejected fæces just before death. A similar case is recorded by Ambrose Paré (*Des Plaies en particulier*, xxxii). An officer, at the siege of Rochelle, received a musket shot, which, entering near the xiphoid cartilage, traversed the diaphragm. After eight months, he died of colic; and on dissection, the colon was found to have passed into the thorax, although the orifice in the diaphragm would scarcely admit a little finger. Littre (*Memoires de l'Acad. des Sciences*, An. 1706) communicates an example of this curious transposition, observed on the body of a dog, wherein the stomach and duodenum had been protruded through the œsophageal opening of the diaphragm.

An infant may even be born with this muscle unnaturally perforated; and hence the thorax be filled with the abdominal viscera. On this principle may be explained a case, which was communicated to Helvetius by Dr. Chauvet of Toulon (*Mem. de l'Acad. des Sciences*, An. 1729). The body of an officer, who had died after long suffering, being opened, the stomach was found in the left thoracic cavity, into which the spleen had also in part escaped by a distinct opening of the diaphragm. The colon had passed into the chest by a third orifice, and re-entered the abdomen by a fourth. From the smoothness and equality of the margins of these perforations, it may be inferred, that they were congenital, and that the transposition of the viscera was the result of primitive conformation.

But it is more particularly the object of M. Percy to treat of the spontaneous and accidental rupture of the diaphragm, of its causes, signs, and the almost invariably fatal consequences to which it leads. These points he professes to develope by the following observations.

A young lady, while endeavouring to suppress her cries amid the pains of child-birth, saw her husband, who was seated near the fire, fall backwards. She uttered a plaintive scream, faltered out a few words, and instantly expired, giving birth to a healthy boy. On dissection, the diaphragm was seen ruptured in shreds, to the extent of five inches, in its left fleshy portion. Two thirds of the stomach, with the omentum and a loop of the colon, had already escaped into the thorax. A little blood was effused into the abdomen, and on the ruptured side of the diaphragm.

Several causes evidently concurred in the production of this accident:—The excessive efforts of child-birth; the simultaneous contraction of all the abdominal muscles; the state of tonicity of the diaphragm; the forcible compression of the abdominal viscera against it by the uterus; a long and violent inspiration; the distension of the lungs by retained air, and the exertions to suppress every expression of pain; and, lastly, sudden terror inflicting a terrible shock upon the diaphragm. The face of this unfortunate woman preserved, after death, an expression of the severest sufferings and agitations to which the mind and body can be subjected. The mouth was hideously distorted, as though the muscles of one side had been paralysed. It will presently be seen, that, in most persons dying from rupture of the diaphragm, the risus sardonicus exists, especially if the injury have taken place in its tendinous centre.—A man died suddenly while vomiting, with extreme difficulty, some gross aliment which he had gorged. Budaëus found, on opening him, a stellated laceration of the tendinous portion of the diaphragm; and the stomach and omentum filling up the solution of continuity, protruding into the thorax, and dragging after them the other viscera. The mouth was prolonged on each side, and exhibited some appearances of a convulsive laugh.—Another man, who had taken a violent emetic for the cure of colic consequent on intemperance, was seized with convulsions, and almost instantly expired. The diaphragm was found, by St. André, ruptured just where the intercostal nerve passes from the thorax to the abdomen; and a portion of the omentum, colon, and pancreas in the left thoracic cavity, where a lesion of some of the vessels of the latter had caused a considerable effusion of blood. Lieutaud saw the diaphragm ruptured, and a portion of the stomach protruded through, in the form of hernia, in a man, of fifty years, addicted to drinking, and subject to rheumatism, who had died in a sudden attack of violent vomiting. But it is to be regretted that, in these cases, nothing is said respecting the *facies* of the deceased, after a kind of death which commonly leaves traces almost characteristic of the accident.

In 1788, the driver of the Diligence from Paris to Calais, while changing horses at Bethune, fell from the roof of the vehicle, crosswise, on the fore wheel, and thence to the pavement. He died almost instantly. M. Percy, on examining the body, was surprized at the great flattening of the superior part of the abdomen. Observing also a sort of smile on the countenance, he predicted that the diaphragm was ruptured, and the thorax filled by the abdominal organs. It was in fact so: the rupture extended, in a crescent form, from the sternum to the tendinous centre. Nearly all the floating viscera of the belly had passed through, and about six pounds of blood were effused into its cavity. The abrupt compression of the abdomen during inspiration, and the sudden and violent impulse of its viscera against the tense and resistent diaphragm, were probably the causes of this rupture. But three signs are here worthy of note; one of which only has been mentioned, and that cursorily, in the preceding cases. These are the convulsive laugh, the dilatation of the thorax, and the sinking in of the abdomen. This last sign, and the second perhaps also, was presented by the body of a man who died suddenly almost under the eyes of Abraham Vater. But he neglected to ascertain the source of this striking appearance.

Among the bodies of the Prussians killed in the night attack of the fort of Bitche, in 1793, M. Percy remarked one with a smiling countenance and eyes yet open. He had been precipitated from the summit of a high rock, and probably fallen on his feet, as both were dislocated. On examination, the abdomen was rather flattened, and the thoracic parietes unusually round and prominent for the figure of the man. The diaphragm was ruptured in every direction; and the cavities of the thorax, particularly the right, filled with a great mass of abdominal viscera. The mediastinum was detached, or rather torn, from the sternum to the extent of four or five inches. A similar and equally curious case is recorded by M. Godefroy, of Rouen, in Leroux's *Journal de Médecine.*

An athletic German, in assisting two men to lower a large cask of beer into a deep vault, stood below and guided it; while the others, placed above, restrained it with cords from descending too rapidly. The former, apprehensive of being crushed, from an undue relaxation of the ropes, made an effort to support the cask himself, and instantly fell senseless to the bottom of the vault, from whence two or three loud sighs were heard to issue. A report immediately got abroad that the vault contained mephitic air, and although four men had without injury descended into it to remove the body, some enlightened physicians had directed vinegar to be thrown in, and fumigations of gun-powder to be employed. On first arrival at the spot, M. Percy thought that the man had been asphyxiated. His face was of a violet hue; the temporal and jugular vessels very prominent; the eyes open and almost projecting from their orbits; the mouth covered with a bloody froth, and blackish blood issued from the nostrils: in fact, the body presented all the characters of asphyxia. Yet, on learning the fact of the four men having descended into the vault with impunity, and farther observing the three symptoms, which have just been mentioned as distinguishing the ruptured diaphragm (although the convulsive laugh is sometimes seen in asphyxia), M. Percy was inclined to refer the death of the man to a cause which his colleagues would not admit, until fully substantiated by dissection. When the thorax was opened, the stomach escaped with noise and impetuosity. It contained an acescent, offensive, red, frothy fluid, composed, for the most part, of beer, and of blood furnished by some ruptured gastric vessels. Beneath this organ were almost all the intestines, the spleen, omentum, and mesentery. Thus, the abdomen had been rendered so flat and flaccid, that the vertebral column might be readily felt through its anterior parietes. The diaphragm was ruptured in its anterior part, semi-circularly, and according to the direction of the ribs, from which it seemed to have been simply torn. Hence the sudden and collective contraction of all the muscles had been capable of bursting the diaphragm, the force of which was inadequate to sustain so powerful an impression: and many, doubtless, have perished in this manner, where no such cause of death has even been suspected.

Peyrilhe mentions that he had seen, in Provence, a mule drop and expire in useless efforts to extricate a carriage stuck fast in the mud. On dissection, the abdominal viscera were seen protruded through the ruptured diaphragm. This accident frequently occurs in beasts of draught, but is almost always mistaken by veterinary surgeons. An instance of it was observed by two medical gentlemen, in a horse which had been purchased by M. Percy, and died suddenly from being over-driven: while the men, who had been employed to investigate the cause of death, merely reported the existence of obstructions in the belly, of worms in the liver, and of gangrenous spots on the intestines. Another example, precisely similar, was lately seen by M. Percy in the streets of Paris. A fine draught-horse, on being struck in the moment of violent exertion, fell suddenly dead: a little blood oozed from the mouth and nostrils. The abdomen was flattened, and the thorax enormously distended. The diaphragm was ruptured from the brisket to the spine, and the whole stomach, half full of food, had passed into the thorax. The flow of blood had proceeded from a laceration of the lungs.

The possible and probable causes of rupture of the diaphragm are too numerous to be all detailed. But to those already mentioned may be added others, which may be deduced from cases resting on the sure basis of anatomical inspection. During the march of the French army upon Trèves, in the commencement of the war, the body of a young Austrian, yet warm, was found upon the road. The eye-lids were half-open; the commissures of the lips drawn back towards the ears, so as to expose the teeth of both jaws; the nose bloody: and death seemed to have been preceded by the vomiting of a green matter. The expectation of finding ruptured diaphragm was realized by dissection. The right side of the chest was filled by a mass of stomach and intestine, which pushed towards the left of the lung and mediastinum, and apparently enveloped the heart. It was subsequently learned from the Austrian prisoners, that this young man had been struck on the breast by the corporal, with the butt end of his piece, and had shortly afterwards expired.

Thus, also, from a blow on the back, died a woman in the sixth month of pregnancy; whose body was examined by Bohn, in 1705. No external lesion, except some traces of redness below the scapulæ, was visible. The diaphragm was ruptured to the extent of four inches. Nothing, however, is said of the passage of the abdominal viscera into the thorax, or of the state of the face. But Bohn adds, that several persons have died suddenly from percussion of the back, in whom, if their bodies had been attentively examined, rupture of the diaphragm would have been found.

Hitherto we have been contemplating this r pture as an accident promptly fatal, and the reality of which can only be ascertained after death. It is lamentable that no example of cure can be opposed to the dark catalogue just traced. Such, doubtless, exist; but they are less numerous and pregnant with consolation than might be wished. The following is taken from Dessault's Journal: A carpenter, aged 39, fell from the hospital of invalids, and was several times, ere he reached a bed of rubbish, caught by the scaffolding. His life was long despaired of. Yet, in six months, he resumed his labour, although teazed with frequent cough, dyspnœa, and increasing pain in the left side of the chest. A fortnight afterwards, in this unfavourable state, he had another fall from a height of twenty feet on the same side. On being conveyed to l'Hotel Dieu, fracture of the seven lower ribs was discovered, with trifling emphysema of the part. He was restless and agitated. His eyes were haggard. He, by times, spat blood; and had short and laborious respiration. His pulse was frequent and high; and he vomited the fluids which, by extreme thirst, he was incessantly constrained to take. Yet he was beginning to mend; when a fall from his bed, on the third night of his admission into the hospital, reproduced all the symptoms, and destroyed him in a few hours. Externally, the abdomen was depressed, without any trace of contusion. Internally, a little blood was effused on the right side. The stomach and arch of the colon had passed into the chest, and occupied nearly the whole of its left cavity: and thus, without any morbid change, the corresponding lung had been depressed and reduced to a very small volume. The heart was pushed to the right; the transit of the viscera into the thorax had taken place by an old opening of the diaphragm, of an oval figure, situated at the exterior of the tendinous centre. It measured two inches and a half in its greatest diameter, and had rounded margins, three or four lines in thickness, to which adhered, towards the chest, the omentum; and the spleen by one of its borders, towards the abdomen. The diaphragm was higher, and more towards the left than ordinary. It had a more recent opening, of three inches in extent, which was traversed by another portion of the colon. The stomach, in the thorax, had its greater curvature upwards, turned towards the mediastinum. The œsophagus was curved. The arch of the colon adhered on one side to the small curvature of the stomach: on the other, it reposed loose upon the diaphragm.

When rupture of the diaphragm is not directly fatal, as rarely happens, it sometimes induces disease more dreadful than death itself. Habitual feelings of distress, obstinate constipation, frequent syncope and vomiting, constant pains in the thorax and epigastrium, and colics, are the symptoms commonly experienced by those who have survived this dreadful accident; as may be seen by reference to the writings of Bartholin, Becker, Sennertus, Fabricius Hildanus, and the Berlin Memoirs.

From these authors, it appears, that the individuals, whose history they have recorded, have all dragged on a wretched existence but for a few months or years after the rupture of the diaphragm; and that, on dissection, the margins of the orifice have been seen rounded, callous, and in a state of cicatrization.

It seems probable, that in those upon whom, after death, the b-dominal viscera have been found situated in the chest, the transposition has been effected before birth, either in consequence of original malformation of the diaphragm, or by the apertures giving passage to the œsophagus and vena cava; both of which have been seen by Schrober and Morgagni so much dilated as to allow a free communication between the two cavities.

We may conclude, that the medical student, whose case Clauder has reported (*Ephemer. Curios. Natur.*); that the woman of whom Jaquitz speaks in the Berlin Memoirs; the youth whose history has been published by Rivière; the girl, of six months, respecting whom an interesting recital may be seen in the Philosophical Transactions—in fact, that all the individuals in whom, after a short life of suffering, the diaphragm has been found perforated, and the abdominal viscera occupying the thorax, have had this defect at birth, or contracted it soon after by violent crying, or at a more advanced age, in consequence of an inflammatory affection of the diaphragm itself, or of some adjacent part.

---

### *Induration and Contraction of the Stomach.**

Dr. Beyerle, of Manheim, has recently found the following morbid appearances on dissection of a female, of sanguineo-choleric temperament, and aged 52. She had borne three children: the abdomen was much distended with water; the intestines empty; the stomach contracted, and drawn towards the diaphragm. Below its right extremity was seen the indurated pancreas. This gland, the stomach, and transverse colon, adhered together. On removal of the stomach, a very strong contraction was discovered in its centre. The induration of the stricture was greatest in the small curvature. The pylorus and cardia were sound. The parietes of the stomach were, in many places, several lines thick; and there the internal membrane was covered with tubercles. The spleen was shrivelled; and the vasa brevia large but short, so that there was scarcely any space between it and the stomach.

This woman, it seems, had frequently complained, from her youth, of stomach-ache, particularly after cold. Emetics and bitters had constantly removed it. About her 49th year, the menses became irregular, and then the gastric affection increased in violence and duration. An emetic, now taken, produced five days' continual vomiting, which was difficultly suppressed. The complaint was at length somewhat relieved by light diet and good management. Menstruation ceased at the 51st year; but, at the

---

* *Beobachtung einer Verhärtung und Verengerung des Magens, &c.* Von Dr. F. J. Beyerle, &c. *Journal der Practischen Heilkunde, &c.* Marz. 1816.

usual periods of its recurrence, the symptoms were invariably aggravated for some days. Three months before death, during a violent attack of vomiting, she was first visited by Dr. Beyerle. The epigastrium was so tense and sore, that accurate examination was impracticable. For some weeks, a mixture of carbonate of magnesia and ox's gall was productive of relief: but this losing its effect, and the paroxysm recurring at the usual period, pills, composed of soap, conium, antimony, gum galbanum, and ox's gall, were substituted for it with evident advantage. From the circumstance of little induration being found on external examination, and the patient taking light food with an appetite, her disease was considered to be merely irritability of the stomach. The amendment was soon interrupted by a return of the symptoms. Oxide of bismuth, however, somewhat allayed the violent vomiting. Opium, at first employed to relieve pain, was afterwards, on account of the existence of obstinate constipation, exchanged for extract of henbane. But the impossibility of a favourable issue became daily more apparent. The patient now rejected medicine, and took only a little milk to slake her urgent thirst. General emaciation suddenly ensued; the feet began to swell; the abdomen was distended; and the woman sunk under constant sense of suffocation, violent pain, and exhausting diarrhœa.

This case, although somewhat more complicated, closely resembles that of central stricture of the stomach, published, by Dr. Palmer, in the first number of our Journal. But, from an engraving which accompanies the description of the German writer, the gastric stricture, in the present instance, appears to have been much less strongly marked than that which the body of the English lady exhibited, and which separated the stomach into two distinct portions, communicating by a small orifice.* This curious lesion of the stomach has very rarely been observed.

---

### *Mercurial Salivation successfully employed in chronic Vomiting from Induration of the Stomach and its Orifices.*†

The effects of mercury, judiciously administered in pyrosis, and other diseases connected with a vitiated state of the secretions of the stomach, experience has long since taught us to appreciate favourably. But we have rarely seen benefit result from its employment in induration, scirrhus, or any of the varieties of morbid structure which this viscus is seen to exhibit. Indeed, in such deplorable cases, this remedy, especially when prescribed in its more active forms, has seemed to exert rather a pernicious than a salutary in-

---

* See *Medico-Chirurgical Journal,* vol. i. page 10.

† *Glücklicher Versuch mit dem Gebrauche des Quecksilbers bis zur Salivation bei chronischen Erbrechen von Verhartung des Magens und des Magenmundes. Von Dr. Christian Berlyn, &c.—Journal der pratischen Heilcunde,* December, 1815.

fluence. Whether the case, which we are about to present from the practice of Dr. Berlyn, were absolutely one of organic lesion of the stomach, may be fairly doubted. The habit, however, of substituting opinion for fact is not confined to Germany. The detail, at all events, is interesting, and will repay us for the labour of recording, if it lead to the successful administration of mercury in but one solitary case of confirmed gastric affection, previously abandoned as hopeless.

A cooper, aged 45, originally healthy, had been attacked, about his 35th year, with intermittent fever. It continued ten weeks, and left him long incapable of working. His digestive powers were particularly enfeebled. He could no longer take the nutritious aliment to which he had been accustomed; and, in a few years, nausea and watery eructations were provoked by the lightest food. The complaint increased, and the most digestible substances were daily rejected by vomiting. Various medicines were tried during six years without relief. The vomiting, now very painful, came on twice, and subsequently four times, a day. There was great emaciation. In this state Dr. Berlyn first saw the man, and prescribed the following pills: ℞. *Sapon. Venet.* ℥ß; *mercur. dulc. (subm. hydrarg.)* ʒj; *opii pulverati* ℈j. The mass was to be made into three-grain pills, two of which were to be taken every morning, and three every night. From July the 29th, when the medicine was first used, the vomiting ceased. On the 3d of August, constipation existing, some indurated fæces were evacuated by a glyster; and the epigastric distention, of which the patient had complained, was thereby removed. Salivation declared itself rather severely on the 4th, and the man felt very feeble. The pills were now exchanged for a tonic mixture, and strong broth and wine were directed. On the 10th, the vomiting returned; and, although the salivation yet continued, the pills were resumed to the number of three daily. Thus the vomiting and gastric pain again gave way; and, singular enough, the salivation almost entirely ceased. By the 14th, the appetite was restored. On the following day, the salivation re-appeared; but the general state of the patient continued favourable. From this period, his amendment was progressive, and he soon completely recovered.

---

### *Efficacy of Moxa, applied to the Vertex, in the Cure of Chronic Head-ache.*

The Moxa is a remedy which, if we mistake not, has hitherto been rarely employed, and is, indeed, very little known by the professional men of this country. We have already noticed the favourable effects of its application in morbid affections of the joints, and in neuralgia of the spermatic cord.* M. Morel,†

---

* See our Review of Roux's Narrative, *Medico-Chir. Journal,* vol. ii. page 56; and our Selections, vol. iii. page 72.

† *Gazette de Santé,* Mars, 1816.

surgeon of Lyons, states that he has employed it with signal and permanent benefit on the epigastric region, for the cure of chronic enlargment of the liver, which, during five years, he had himself laboured under. It was consequent on two attacks of quartan fever, had resisted all the ordinary remedies, and reduced him to a state of great weakness and emaciation. The moxa was thrice applied; the last time after a lapse of several years. The case has been published at length in a contemporary Journal; hence, we may be excused from detailing it more fully here.

We have now to present an example of the successful application of this remedy in a chronic affection of the head, by Dr. Duparque, an estimable French writer.* The subject of the case was an unmarried female, of nervous temperament and feeble constitution, aged 27. Menstruation had gone on with trifling derangement. The pain affected the whole cranium, particularly the forehead, and was constant, but varied in intensity. It had existed eight years, and was so violent as to deprive the patient of sleep. Frequent epistaxis, both in the intervals of menstruation and during the flow, was productive of no marked relief. There were occasional flushings of the face; the capillary system of which was so much developed as to exhibit a deep blush on the slightest moral or physical impression. In May, 1813, all the impending symptoms of phthisis had been, for some time, announced: oppression, cough, glairy and puriform expectoration, partial night sweats, and loss of voice. The pulse was small, wiry, hard, and frequent. Topical application of cold, leeches to the ankles and vulva, general bleeding, stimulant pediluvia, antispasmodics, and henbane, were prescribed without benefit. In June, there was an increased rush of blood towards the head, signalized by flushed face, injected conjunctiva, and noise in the ears. The pain, in July, became intolerable, accompanied with drowsiness, which was interrupted, every three or four seconds, by a sense of pulsation in the brain. This dreadfully aggravated the pain about the saggittal suture, and acted on the patient like an electric shock. Her intellect was unimpaired. The forehead was hot; the cranial integuments sore about the saggittal suture. The countenance was expressive of suffering. The continued application of ice to the head, blistering, and the liberal use of musk, did nothing. The symptoms increased in violence. The convulsive shocks were now so strong as to shake the bed. The eyes were fixed, and their pupils dilated: the jaws clenched as in tetanus. The pulse was small and fluttering; the abdomen tense, painful, and constipated. Baths and emollient glysters were useless. The case was now considered desperate; and, as a last effort, the moxa was, on the 10th of July, applied about the middle of the sagittal suture,

---

* *Observations sur une Chephalalgie chronique, guérié par l'application d'un moxa au sommet de la tête. Par M. Duparque, D. M. Bulletin de l'Athénée de Médecine de Paris, Avril,* 1817.

where the integuments were tender and somewhat puffy. The eschar being too small and superficial, a larger was produced by a repetition of the moxa. Onthat evening there was a marked mitigation of the symptoms. In about four days, sleep and appetite began to return. In proportion as suppuration came on, the health of the young woman improved, and was soon perfectly re-established. The abdominal pain and tension yielded, meanwhile, to the influence of baths and fomentations. The strength was recruited by tonics; and on the 21st of August, she was dismissed quite well, with the exception of some of the phthisical symptoms which she had long exhibited.

In another of the French Journals, there is a case detailed by a Physician of Brussels, exhibiting the efficacy of the moxa in severe pulmonary hæmorrhage.* The patient, aged 43, was a military officer; who had honourably distinguished himself, and been dreadfully mutilated in the French service. His thorax had been traversed by a musket ball. Demulcents, pediluvia, asses milk, and Iceland moss, were productive of benefit; but, from the hectic-like and emaciated state of the patient, his case was deemed hopeless. Under these circumstances, the moxa was applied on the posterior inferior part of the thorax, in two places, six inches distant; and a seton was passed from one to the other. Scarcely had suppuration commenced, when the breast was relieved. Baths, with the lichen, were employed. In two months the seton was withdrawn: and, two years afterwards, the officer remained perfectly well.

We shall be glad to receive from our Correspondents a detail of their experiments with the moxa.

---

### *Case of Croup, in which the Operation of Tracheotomy was unsuccessfully performed.*

Our readers are aware that this operation has been practised by English Surgeons, in various affections of the larynx and trachea, with complete success.† Dr. Hervez, it appears from the following history,‡ has been less fortunate in the attempt. The case, we think, displayed the characters more decidedly of laryngeal, or even pharyngeal, than of tracheal inflammation.

A girl, of six years, had been twelve days ill when first visited by Dr. Hervez. The symptoms were those of croup, with swelling of the submaxillary glands and tonsils. The latter were co-

---

* *Heureux effet du moxa dans une affection très-grave de la Poitrine. Gazette de Santé, Avril* 1817.

† See a Case by Mr. Chevalier, in the 6th vol. of the *Medico-Chirurgical Transactions;* and an excellent paper by Mr. Lawrence in the same work and volume.

‡ Observation sur une angine membraneuse, pour laquelle on a pratiqué infructueusement *l'opération de la trachéotomie. Gazette de Santé,* August, 1817.

vered with a whitish film, and presented a sloughy point, by which the breath was rendered offensive. The child was sinking. A cough, characteristic of an affection of the larynx, was heard at rare intervals. The obstacle to respiration seemed to exist rather in the posterior fauces than in the larynx. An emetic, sinapisms to the feet, and blisters, with the internal use of wine, were prescribed. On the third morning, Dr. Hervez, summoned in haste, found the child with a livid countenance, frequent respiration, and slow feeble pulse, He immediately proposed an operation as the only resource. M. Chomel, whom he had called to his assistance, wished first to try blistering and the sulphuret of potash ; but the symptoms became too urgent to admit of delay.

A small arterial branch and two or three small veins sprang, and were tied successively ere the trachea was cleared. The membraneous space, separating the second and third cartilaginous ring, was exposed, and freely cut. The air immediately escaped, driving before it a bloody froth. The child was laid on her side, with the head low; and respiration became more free. Scarcely had success been anticipated, when suddenly the air ceased to pass through the orifice. On being vertically enlarged by incision of the third cartilaginous ring, air rushed out anew; then suddenly stopped again; and the stream could not be re-established even by the employment of a canula. The child shortly afterwards expired. The operation had frequently been embarrassed by the flow of blood; a little of which was, it seems incorrectly, supposed to have got into the trachea. Proper attention to the ligature of every vessel as it sprang, and to the position of the patient, would obviate all danger from this source.

On dissection, the whole pharynx, posterior part of the velum palati, and tonsils were covered with a white, thick, adventitious membrane, readily separable. Gangrene had commenced in the left tonsil. The internal cavity of the larynx was nearly obliterated by tumefaction of the sub-mucous cellular structure. The adventitious membrane was here strongly marked, and detached with facility. It was less thick than that of the pharynx, and terminated at the place where the opening into the trachea had been made. By this membrane the orifice had been obstructed. The trachea and bronchiæ, although of a bright red colour, exhibited not the slightest trace of the membraneous formation. Hence the case was a very favourable one for the trial of tracheotomy,

---

### *Observation on the Employment of Cupping Glasses in obstinate Diarrhœa.*

The following Case, by Dr. Lacombe, affords a tolerably correct specimen of French practice in intestinal affections. To the generality of British readers such a remedy will, doubtless, be novel. Properly viewed, however, its efficacy is calculated to suggest an useful reflection.

A girl, of lymphatic temperament, aged 10, had suffered, for one week, from catarrhal diarrhœa, attended with colic, abundant mucous evacuations, irritative fever, evident loss of flesh and strength. Rice-water with gum, opiates, injections composed of decoction of poppies and flour, after each evacuation, and abdominal frictions, every six hours, with ammoniacal liniment, were tried, for four days, without effect. Six cupping glasses were then applied to the integuments in the direction of the arch of the colon, and left on for eight hours. The diarrhœa, from that period, was permanently arrested.

On what principle could dry cupping operate, but on that of deriving the blood from the deep-seated parts to the surface? A disciple of Dr. Hamilton would have effected the cure, in such a case, by a different and perhaps more salutary process. He would have unloaded the gorged intestinal vessels by the active exhibition of calomel, and saline purgatives. EDITORS.

